# A large ongoing outbreak of hepatitis A predominantly affecting young males in Lazio, Italy; August 2016 - March 2017

**DOI:** 10.1371/journal.pone.0185428

**Published:** 2017-11-10

**Authors:** Simone Lanini, Claudia Minosse, Francesco Vairo, Annarosa Garbuglia, Virginia Di Bari, Alessandro Agresta, Giovanni Rezza, Vincenzo Puro, Alessio Pendenza, Maria Rosaria Loffredo, Paola Scognamiglio, Alimuddin Zumla, Vincenzo Panella, Giuseppe Ippolito, Maria Rosaria Capobianchi, Gruppo Laziale Sorveglianza Epatiti Virali (GLaSEV)

**Affiliations:** 1 Dipartimento di Epidemiologia Ricerca Pre-Clinica e Diagnostica Avanzata, National Institute for Infectious diseases Lazzaro Spallanzani, Rome, Italy; 2 Department of Infectious Diseases, Istituto Superiore di Sanità, Rome, Italy; 3 Azienda Sanitaria Locale Roma 1 Dipartimento di Prevenzione—U.O.S. Controllo Malattie e Gestione Flussi Informativi, Rome, Italy; 4 Azienda Sanitaria Locale Roma 3 Servizio di Igiene e Sanità Pubblica Profilassi delle malattie infettive e parassitarie, Rome, Italy; 5 Division of Infection and Immunity, University College London and NIHR Biomedical Research Centre, UCL Hospitals NHS Foundation Trust, London, United Kingdom; 6 Direzione Regionale Salute e Politiche Sociali, Regione Lazio, Rome, Italy; Fondazione IRCCS Istituto Nazionale dei Tumori, ITALY

## Abstract

The hepatitis A virus (HAV) is mainly transmitted through the faecal-oral route. In industrialized countries HAV infection generally occurs as either sporadic cases in travelers from endemic areas, local outbreak within closed/semi-closed population and as foodborne community outbreak. Recently, an increasing number of HAV infection clusters have been reported among young men-who-have-sex-with-men (MSM).

The Lazio Regional Service for the epidemiology and control for infectious diseases (SeRESMI) has noticed an increase of acute hepatitis A (AHA) since September 2016. Temporal analysis carried out with a discrete Poisson model using surveillance data between January 2016 and March 2017 evidenced an ongoing outbreak of AHA that started at the end of August. Molecular investigation carried out on 130 out of 513 cases AHA reported until March 2017 suggests that this outbreak is mainly supported by an HAV variant which is currently spreading within MSM communities across Europe (VRD_521_2016).

The report confirms that AHA is an emerging issue among MSM. In addition through the integration of standard (case based) surveillance with molecular investigation we could discriminate, temporally concomitant but epidemiologically unrelated, clusters due to different HAV variants. As suggested by the WHO, in countries with low HAV circulation, vaccination programmes should be tailored on the local epidemiological patterns to prevent outbreaks among high risk groups and eventual spillover of the infection in the general population.

## Introduction

Acute hepatitis A (AHA) is usually a self-limiting disease caused by the hepatitis A virus (HAV). HAV is mainly transmitted through the faecal-oral route either by direct person-to-person contacts or by contaminated water and/or food products. The incubation period ranges between 15–50 days (mean 28 days). [1–2] HAV infection resolves in 2–8 weeks, in parallel with the development of humoral and cellular immune response.[3] Natural infection with HAV confers lifelong immunity. [2,3] The diagnosis of AHA is usually based on the detection of anti-HAV IgM; in addition, viral genomes can be easily detected in blood and stools. Infected persons are most likely to transmit HAV from 1–2 weeks before the onset of illness, when HAV concentration in stool is at the highest level. Asymptomatic disease is relatively common in children aged less than 6 years; in contrast, about 70% of adults develop acute hepatitis with increased liver enzymes and often jaundice. [4] In industrialized countries HAV infection generally occurs as either sporadic cases in travelers form endemic areas, local outbreak within closed/semi-closed populations (primary schools, ethnic minority, religious groups, people who inject drugs, marginalized groups) and as foodborne community outbreak. [5] Recently, an increasing number of HAV infection clusters have been reported among young men-who-have-sex-with-men (MSM). [2,6–7]

Since September 2016 the Regional Service for the Epidemiology and Control for Infectious Diseases (SeRESMI) has notice an unexpected increase of AHA in Lazio (central Italy). Hereby we report the results of the molecular epidemiology investigation carried out to confirm a potential outbreak of AHA outbreak, describe temporal trends of incidence and identify chains of transmission and specific group at highest risk of infection in order to implement *ad hoc* public health measures.

## Methods

### Study design

The study is designed as a temporal analysis of surveillance data to assess AHA outbreaks in 3 different population groups (i.e.: men, women and children) and a molecular epidemiology investigation to assess genetic similarities between local HAV isolates and other relevant HAV isolates currently causing outbreak in Europe. The report follows recommendations of the STROME-ID statement. [8].

### Setting

Lazio is an Italian Region with 5,888,472 inhabitants. The city of Rome is the most populated area (2,864,731 inhabitants) followed by the metropolitan area of Rome (1,475,743 inhabitants), the province of Latina (574,226 inhabitants), the province of Frosinone (495,026 inhabitants), the province of Viterbo (320,279 inhabitants) and the province of Rieti (158,467 inhabitants). Men aged ≥ 18 represent 39.77%, women 43.82% and children/adolescents aged 0–17 years 16.41% of the overall resident population.

SeRESMI has been established since 2015 at the National Institute for Infectious Diseases Lazzaro Spallanzani (INMI) in Rome. SeRESMI has been appointed to coordinate the surveillance and control activities of the 10 local heath units (ASL) operating in the Region. On November 2015, the regional heath authority decided to change the notification system for several infections. Since this date new cases of AHA should be notified to the competent health authority as soon as possible, and a serum or stool sample should be sent to the Regional Reference Laboratory for Infectious Diseases, which is also established at INMI.

### Molecular investigation

Molecular investigation has been carried out for all patients with AHA and detectable HAV RNA in either serum or stool until January 2016. From January 2017, only a fraction of samples underwent molecular analysis. The phylogenetic analysis was based on a 460nt sequence encompassing the VP1/2A region of HAV genome.[9]

### Participants and case definition

**Participant:** all new cases of AHA occurred in Lazio between January 1 2016 and March 31, 2017.

**Acute hepatitis**: patient any of the following symptoms: fever, headache, malaise, anorexia, nausea, vomiting, diarrhea, abdominal pain and a) jaundice, and / or b) elevated serum alanine aminotransferase.

**Confirmed case of AHA**: a case of acute hepatitis who tested positive for anti-HAV IgM and/or with detectable HAV RNA in either blood or stools. [10]

**Probable case of AHA**: a case with acute hepatitis and close contact with at least one confirmed case. [10]

**AHA outbreak**: a statistically significant temporal cluster of AHA (p<0.050).

**Monophyletic cluster**: 2 or more cases of AHA infected with HAV variant whose sequence is located in the same branch of the phylogenetic tree, supported by a bootstrap value >80.

### Data source and variables

All data were collected merging the information from the SeRESMI registry for infectious diseases notification with those from the registry of Reference Laboratory.

Data of symptoms onset, gender and age were collected for all AHA cases. Cases were stratified according to 3 different classes of risks, i.e.: men (male subjects aged ≥ 18 year), women (female subjects aged ≥ 18 year) and children/adolescent (subjects aged ≤ 17 years regardless of sex).

### Laboratory methods

Anti-HAV IgG and IgM testing was performed with Architect system (Abbott Diagnostics, Abbott Park, IL, USA). RNA was extracted from serum or stool samples, depending on available material, using QIASYMPHONY automated instrument (QIAGEN, Hilden, Germany). RNA was reverse transcribed, and cDNA was amplified using specific primers for VP1/2A junction. [9] The resulting amplicons were sequenced with Prism BigDye (Applied Biosystems, Forster City, CA, USA) in an ABI3100 DNA automated Sequencer.

CLUSTAL W program was used for multiple sequence alignment. The best fit model was identified and a phylogenetic analysis was performed using the maximum-likelihood method with the Hasegawa-Kishino-Yano model + G, implemented in MEGA6 software. To assess the significance of the nodes, bootstrap analysis with 1000 replicates was performed (>80% considered significant). The phylogenetic tree was made by using a a 460nt sequence encompassing the VP1/2A region of HAV genome. [9] In the phylogenetic tree, all the HAV sequences obtained from cases referred to the Laboratory since 2013 were included. Reference sequences from retrieved from GenBank were included as well. In addition the sequences of four strains, reported to be associated with current epidemic clusters in other European countries have been included in the phylogenetic analysis: VRD_521_2016, first described in United Kingdom, RIVM-HAV16–090 and RIVM-HAV16–069 first described in The Netherlands, and V16_25801, first reported in Germany [7,11–12].

### Statistical analysis

The presence of AHA outbreak was assessed by a discrete Poisson analysis separately for the 3 different populations at risk (i.e. men, women and children) with a 1-month time window. Incidence was calculated using the resident population in Lazio on January 1, 2016 according to official statistics. [13] Expected cases have been calculated using indirect standardization. Relative risk was calculated as the ratio observed/expected cases within the cluster, divided by the ratio observed/expected outside the cluster. Statistical significance of temporal cluster(s) has been assessed by Montecarlo simulation model with 999 iteration. All analysis was carried out with SaTScan version 9.4 [14]

## Results

### Participant and descriptive data

**Fig 1:**
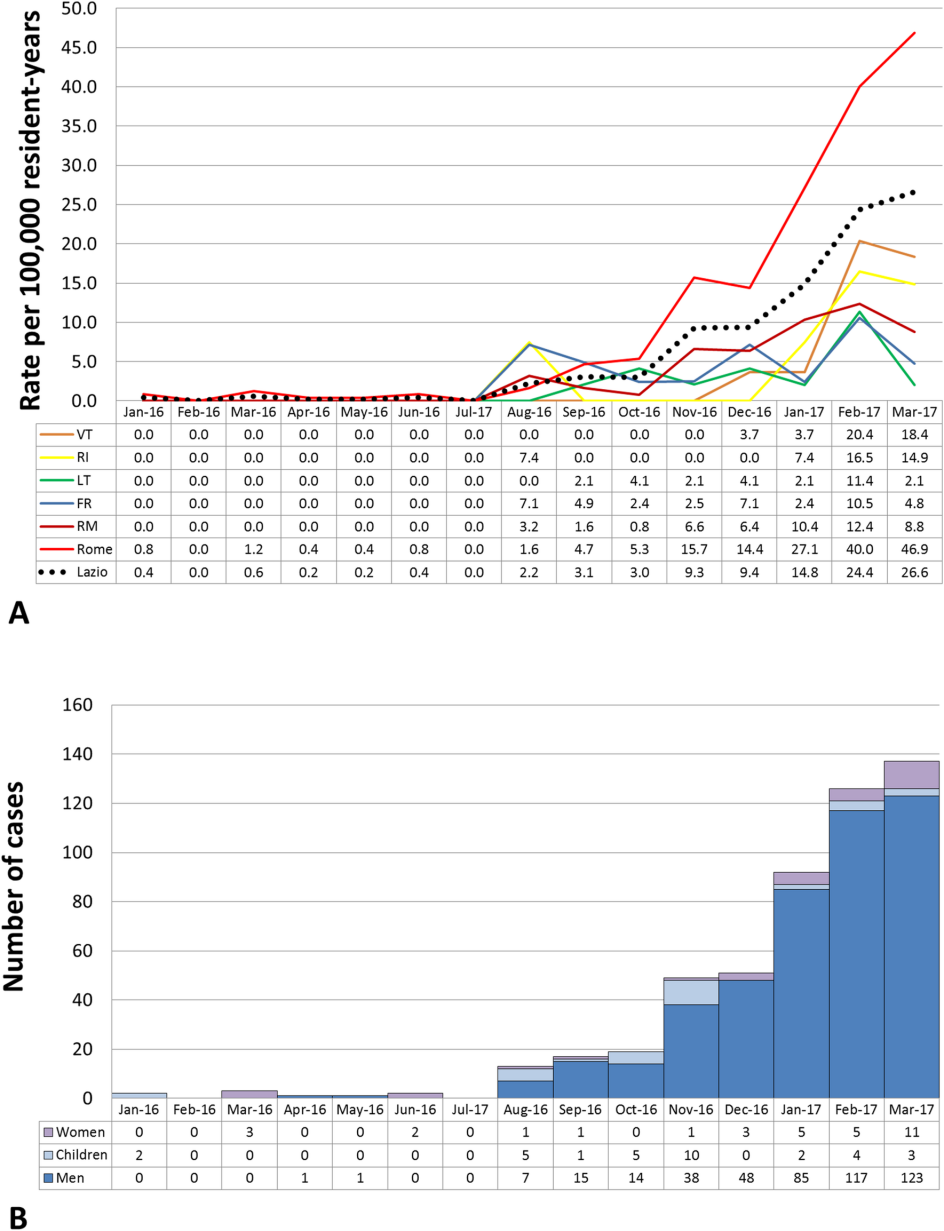
Incidence of AHA cases. A) Incidence of the AHA cases occurred among people resident in Lazio, according to area of residence (i.e. Frosione (FR) 17 cases; Latina (LT) 13 cases; Rieti (RI) 6 cases; Metropolitan area of Rome (RM) 61 cases; City of Rome (ROMA) 377 cases; Viterbo (VT) 12 cases; total cases: 486). Cases occurred in non-resident people (N = 27) were not included. B B) Temporal distribution of the 513 cases of AHA occurred in Lazio between 1 January 2016 and 31 March 2017, according to risk class, i.e. men (N = 449); women (N = 32); children (N = 32).

A total of 513 AHA cases were recorded in Lazio between 1 January 2016 and 31 March 2017. Of them, 449 (87.52%) were men, 32 women (6.24%), and 32 children (6.24%). The median age was 33 years (inter-quartile range [IQR]: 27–41; range 9 months to 84 years). HAV infection was laboratory confirmed in 509 patients, while 4 cases were reported because presenting with acute hepatitis after close contact with a confirmed case (probable cases). Spatio-temporal distribution of cases (Fig 1A) suggests that since August 2016 the risk of infection steadily increased among residents in the city of Rome, where the incidence of AHA increased form less than 2 (January-August 2016) to 46.9 (March 2017) cases per 100,000 resident-years. The distribution of AHA cases according to month of symptoms onset and class of risk (i.e. men, women or children) is reported in Fig 1B.

### Outcome data: Temporal analysis

Temporal analysis provided strong evidence that statistically significant clusters of AHA occurred at different times for either men, women or children (Table 1).

**Table 1:** Temporal cluster analysis.

***Panel A*.** *Population Parameters*	*Men*	*Women*	*Children*
Time window	01/01/2016-31/03/2017		
Resident population	2,341,883	2,580,432	966,157
Number of cases	449	32	32
Annual rate per 100.000 inhabitants	15.4	1.0	2.7
**Panel B.** *Outbreak parameters*	*Men*	*Women*	*Children*
Cluster duration	Nov 2016 to Mar 2017	Dec 2016 to Mar 2017	Aug 2016 to Nov 2016
P-value	0.001	0.001	0.001
Observed cases	411	24	21
Expected cases	148.68	8.49	8.56
Annual rate per 100.000 inhabitants	42.5	2.8	6.5
Expected/Observed ratio	2.76	2.83	2.45
Relative Risk	21.85	8.31	5.23

The largest cluster was observed among men, and includes 411 cases of AHA with a median age of 34 (IQR 28–41). It could be appreciated since August 2016, and became statistically significant since November 2016, being still ongoing at the time of the analysis. The incidence in this cluster was remarkably high (42.5 cases per 100,000 resident-years), accounting for a relative risk (RR) of 21.65 compared to the period January-October 2016).

A second cluster was found among women, and includes 24 cases of AHA with a median age of 37 (IQR 27–50). It became statistically significant since December 2016 and, similarly to the cluster among men, it is still ongoing. The women cluster was featured by an incidence much lower than that observed in men (2.8 cases per 100000 resident-years), but yet with a RR of 8.31.

A third cluster was found among children/adolescents, and includes 21 cases of AHA with a median age of 7 (IQR 4–11). It occurred from August to November 2016 and is currently expired. The incidence in this cluster was 6.5 cases per 100000 resident-years, with a RR 5.23.

### Main results of molecular analysis

**Fig 2:**
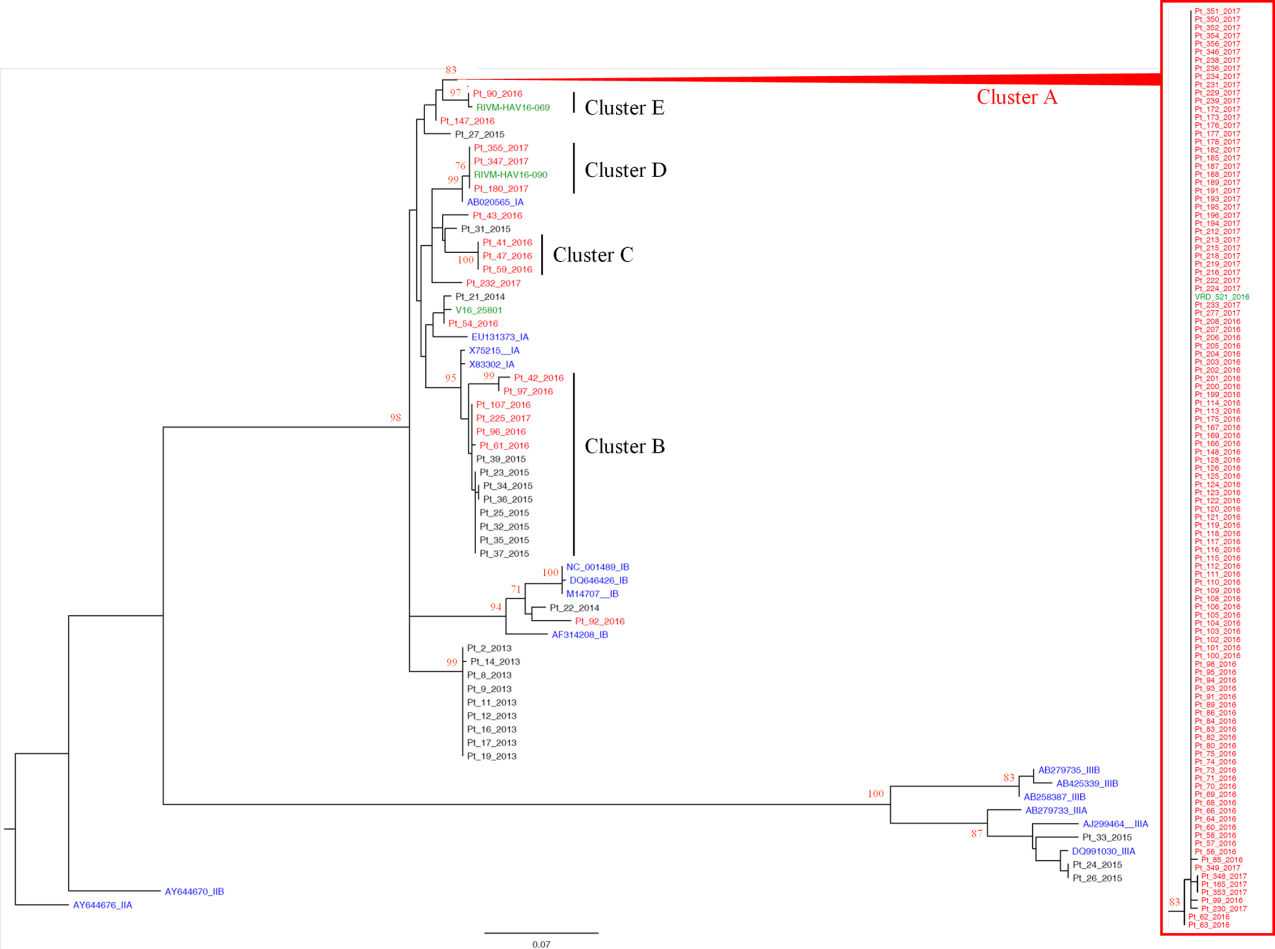
Phylogenetic analysis. Phylogenetic tree, built with a total of 174 460nt-long sequences encompassing the VP1/2A junction region of HAV genome, based on the maximum-likelihood method with the Hasegawa-Kishino-Yano model + G. All the sequences obtained in 2016–2017 from Lazio region (N = 130, patient number, in red) are included. In addition, HAV sequences from Lazio cases referred to the Laboratory from 2013–2015 (N = 24, patient number, in black) are included. The tree also includes 16 reference sequences from GenBank (genotype IA: X75215; EU131373; AB020565; X83302; genotype IB: M14707; DQ646426; NC001489; AF314208; genotype IIA: AJ644676; genotype IIB: AY644670; genotype IIIA: AJ299464; DQ991030; AB279733; genotype IIIB: AB279735; AB425339; AB258387, in blue), and the 4 sequences (VRD_521_2016 and RIVM-HAV16-90, RIVM-HAV16-69 and V16_25801, in green) recently reported to be associated with epidemic clusters among MSM in other European countries (in blue). One genotype IIA sequence (AY644676) was used as the outgroup. The bar represents the genetic distance (substitution per nucleotide position). Bootstrap analysis with 1000 replicates was performed to assess the significance of the nodes; values greater than 80.

The molecular analysis included 130 out of 513 cases of AHA reported since January 2016 to March 2017; the phylogenetic tree included all sequences of HAV isolates from Lazio region since 2013 (n = 24), and 17 reference sequences available in international databases. Among these, four sequences (VRD_521_2016, RIVM-HAV16-069; RIVM-HAV16-090 and V16_25801) represented HAV molecular variants currently circulating in Europe among MSM. The analysis showed the presence of 5 contemporary clusters identified with the letter A to E in Fig 2.

Cluster A (HAV subtype IA) included 112 cases, of which 106 were men, 5 women and 1 male adolescent aged 17. This cluster includes also the VRD_521_2016 variant firstly identified among UK MSM on July 2016. This variant was never reported in Italy before and was firstly identified in Lazio at the end of August 2016.

Cluster B (HAV subtype IA) included 6 cases from 2016–2017 (1 men, 3 women and 2 children). This variant has no relation with other variants associated to infection among MSM in Europe. HAV variant genetically similar to cluster B was already present in Italy and was associated to foodborne infections since 2015 (8 cases from 2015 included in the tree).

Cluster C (HAV subtype IA) included 3 cases (2 women and 1 children) returning from Romania were a large outbreak was ongoing. It has no relation with other variants associated to infection among MSM in Europe and was never found in Lazio before.

Cluster D (HAV subtype IA) included 3 men with symptom onset between 10 December 2016 and 16 March 2017. This cluster also includes the RIVM-HAV16-090 variant, firstly identified among MSM who participated to EuroPride held in Amsterdam on July 2016. This variant was never reported in Lazio before.

Cluster E (HAV subtype IA), included 1 man who developed symptoms on 6 September 2016. This HAV isolate was associated to variant RIVM-HAV16-069, circulating among MSM in UK and Nederland. Also this variant was never reported in Lazio before.

Five sequences (4 HAV subtype IA and 1 HAV subtype IB) from 2016–2017 cases are not included in any of these clusters; however, one of them (Pt_54_2016) is close to the variant V16_25801, although clustering is not statistically supported.

**Fig 3:**
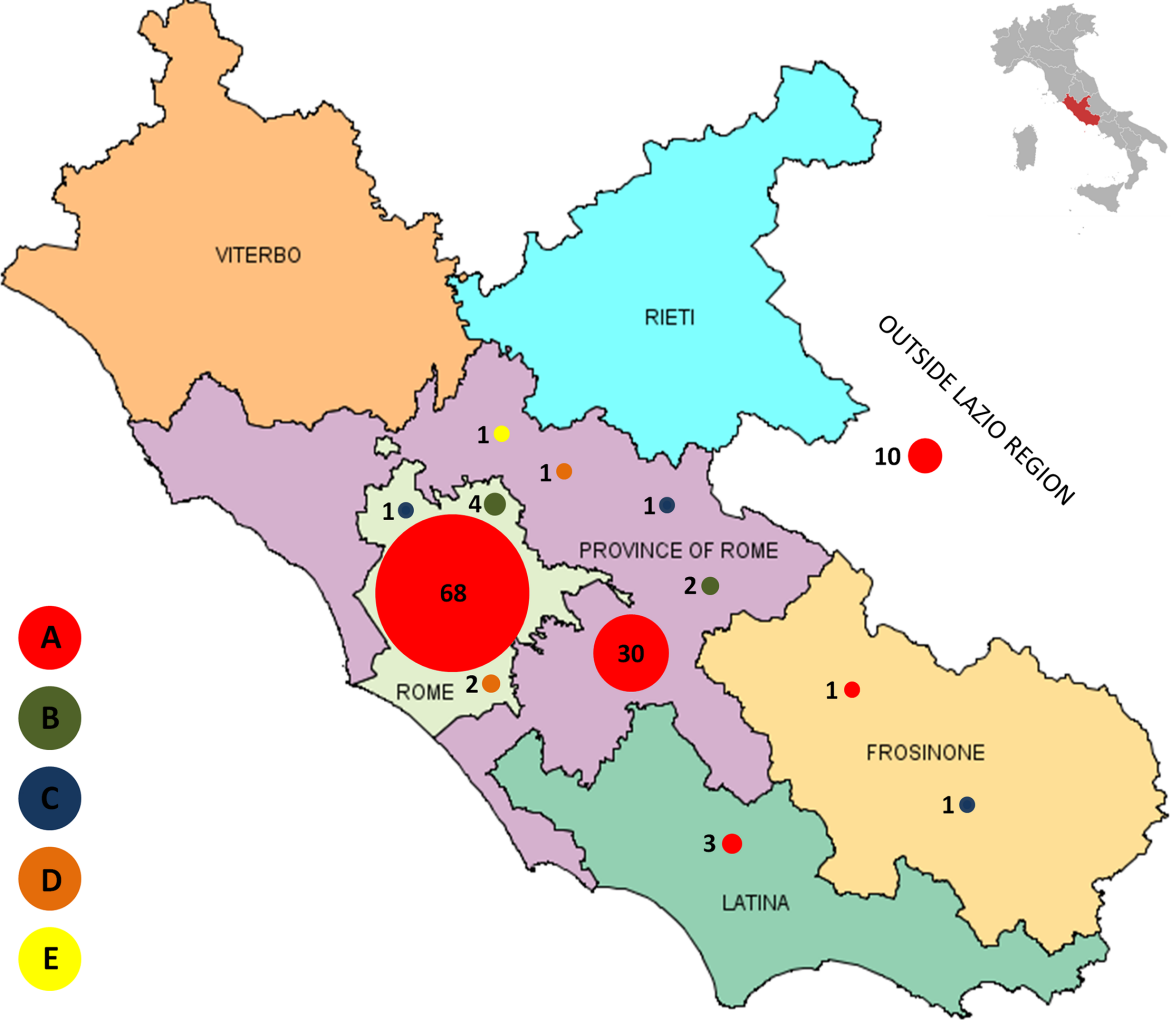
Geographical distribution of HAV isolates. The figure describes the distribution in Lazio of the 125 HAV isolates according to the 5 clusters (i.e.: Cluster A N = 112 -red, cluster B N = 6 -green, cluster C N = 3 -blue, cluster D N = 3 -orange, cluster E N = 1 -yellow). The figure did not include the 5 sporadic HAV isolates.

Fig 3 shows the geographical distribution of the 130 HAV isolates.

## Discussion

### Key results

Our study provides strong evidence that a large AHA epidemic is currently ongoing in Lazio, mainly involving young men living in Rome. The results of molecular investigation, the age and gender of most cases suggest that infections are mainly transmitted by sexual contacts between men. The analysis also identified a temporal cluster among women which most probably represents the result of spillover of the epidemic among women through wider social network. A third temporal cluster was found among children; however, this cluster is not genetically related with the large cluster among men and is currently expired.

### Interpretation

HAV molecular variant belonging to monophyletic cluster A appeared in Lazio concomitantly with the sharp increase of AHA incidence observed since August 2016, and predominantly spreading among young men. This molecular cluster included 106 men, 5 women and one adolescent male with a male to female ratio of 21.2 to 1. This extremely high male to female ratio suggests that sexual practices between male were the primary transmission route of the infection.[15] In support of this hypothesis we found that the HAV isolates associated with cluster A is identical to the HAV variant (VRD_521_2016) which has been firstly identified on July 2016 among UK MSM returning from Spain [7] and it is currently sustaining HAV outbreak in UK, [11] Germany [16] and Israel. [17] The late inclusion of women in this cluster suggests the potential spillover of the epidemic through social networks, possibly involving transmission routes not limited to sexual practices. Besides the molecular variant of cluster A, few men were also infected with RIVM-HAV16-090 (cluster D) and RIVM-HAV16-069 (cluster E) variants. Both variants were associated with AHA in MSM; in particular RIVM-HAV16-090 was firstly identified among MSM who participated in the EuroPride festival in Amsterdam (23 July to 7 August 2016) while RIVM-HAV16-069 was associated in isolate cases of AHA in MSM form UK [11] and Nederland. [18] However, these variants emerged later in time when we started to carry out molecular typing on a proportion of cases, thus the actual relevance of variants associated to molecular cluster D and E could have been underestimated.

The HAV molecular variant associated with cluster B infected 2 children, 3 women and 1 men. Investigation carried out by the local heath unit found that the 2 children belonged to a larger familiar cluster of AHA including 8 additional cases living in the same compound in a Gipsy camp in Rome. All children aged 1–11 years with symptoms onset between 12 October and 17 November 2016. The outbreak was controlled by the intervention of the local health authority which implemented a vaccine programme against HAV including 70 children living in the camp. HAV variant associated with cluster B was endemic in Lazio and already found in a cluster associated with food consumption in 2015.

Cluster C included 3 subjects who had traveled in different times to Bacău County (Eastern Romania) within the incubation period. These cases may represent the importation of a viral variant possibly endemic in the area of Bacău County.

### Generalisability

In industrialized countries, occasions for exposure during childhood are infrequent, thus young adults remain susceptible to infection, favoring the occurrence of periodic AHA outbreaks in selected groups at the highest risk. [2] Italy used to be at high risk of AHA outbreaks, due to the diffuse habit of raw fish consumption. [19] Universal vaccination programme implemented in the most endemic areas of Italy, Puglia, have successfully controlled periodical AHA outbreaks associated to foodborne infections. In these settings, annual incidence of AHA dropped from 14.8 to 0.8 per 100,000 persons-year between 1998 and 2009. [20] At present Italy has a transitional epidemiology for HAV, with some Regions being at very low risk, while other Regions are at low to intermediate risk. [21] According to ECDC classification, [22–23] mean annual incidence of AHA was very low between 2006 and 2015 in Lazio, i.e. about 1.77 cases per 100.000 persons-year (range 0.73–3.09), according to official regional estimates (unpublished data). Nevertheless, local outbreaks have been described as consequence of foodborne infection and/or transmission among special groups. [6,9,24] In particular, AHA outbreaks among MSM seems an emerging issue in Lazio as well as in other European settings.[23] HAV is particularly suited for being transmitted among MSM for several reasons. Firstly, HAV is mainly transmitted by oro-fecal route, a contact which is very frequent during male-to-male sexual intercourse and for which the use of barrier protection (e.g. use of condom) is largely inefficient. Secondly, the virus can be transmitted 1–2 weeks before symptoms onset, thus contagious persons can be hardly recognized. Thirdly, gay oriented sex-venues (e.g. men-only clubs and saunas) usually offer the opportunity to have finger food, thus in these settings costumers may come in contact with HAV either directly, by sexual contacts, or indirectly, through contaminated food or beverages.

A similar situation occurred in 2008, when a large and long-lasting monophyletic outbreak of hepatitis A was reported in the Rome metropolitan area, involving a high proportion of HIV-infected MSM. All the infections detected in MSM were attributable to a monophyletic strain of HAV, circulating also in other parts of Europe around the same period. [6]

According to WHO guidance for areas with low incidence of AHA, [25] in Italy National Vaccine Programme (Piano Nazionale Prevenzione Vaccinale) has been recommending anti-HAV vaccination to several at risk groups including MSM, since 2012.[26–27] To enhance National recommendations in response this outbreak, the Lazio Regional Health Authority has launched, since March 2017 a programme to control viral hepatitis in adults aged 18–45 years. This programme incorporate hepatitis A vaccination in comprehensive set of interventions including screening for viral hepatitis (HAV, HBV and HCV) education for prevention of viral hepatitis and HIV, vaccination for patients who were found susceptible to HBV and an assisted access to regional service for chronic viral hepatitis and HIV care. The programme is currently ongoing and have screened about 900 subjects in the first five months of activity. Since 20 June 2017 we have suspended anti-HAV immunization as the consequence of vaccine shortage. [7] All other components of the programme are still ongoing.

In conclusion our study shown that current HAV outbreak among young males in Lazio is a part of a larger continental outbreak which is spreading among MSM community across Europe. There is a need to promote the access to anti-HAV vaccination in Lazio for MSM. In our opinion the access to vaccine should be at best implemented trough targeted programmes that integrate HAV vaccination within a framework of intervention including measure to control and to estimate burden of viral hepatitis at local level.

### Limitations

The main limitation of our study is that we did not systematically collect information about sexual orientation and travel of AHA cases. Nevertheless, the very high male to female ratio strongly suggests that the outbreak was mainly supported by transmission among MSM. In addition, the same HAV variant is found in currently ongoing outbreaks among MSM in other European cities. Since by January 2017 we stopped to systematically perform the molecular typing of all HAV isolates, the actual weight of the HAV molecular variants associated to monophyletic clusters D (RIVM-HAV16-090) and E (RIVM-HAV16-069) may be underestimated. This decision has been taken as the vast majority of cases at that time were included in the monophyletic cluster A. Therefore, a sample of infected population was considered sufficient to inform about possible epidemiological changes.

## Supporting information

S1 Dataset This dataset contain patients infromation at the time the analysis was carried out (7th April 2017). SERESMI database is daily updated. Current data for HAV may marginally change as the consequence of notification delay. **Variant:** molecular variant of HAV according to the phylogenetic analysis in Fig 1 (letter A-E identify identical clusters, X identify variants outside cluster; na stand for not available). **Month:** month of the years when the case(s) occurred. **Year:** year when the case(s) occurred. **Place:** geographical location of case(s). **Class**: class of case(s) (i.e. male, female or child). **Number**: number of cases with identical feature.
(XLS)

## Abbreviations

HAV: hepatitis A virus
AHA: acute hepatitis A
MSM: men-who-have-sex-with-men
SERESMI: Lazio Regional Service for the Epidemiology and Control for Infectious Diseases
INMI: National Institute for Infectious Diseases Lazzaro Spallanzani
RR: relative risk
iqr: interquartile range

## References

[1] European Centre for Disease Prevention and Control (ECDC). Hepatitis A virus in the EU/EEA, 1975–2014. ECDC technical report. Stockholm: ECDC. 2016. Available from: http://ecdc.europa.eu/en/publications/Publications/hepatitis-a-virus-EU-EEA-1975-2014.pdf

[2] Vaughan G, Goncalves Rossi LM, Forbi JC, de Paula VS, Purdy MA, Xia G, Khudyakov YE. Hepatitis A virus: host interactions, molecular epidemiology andevolution. Infect Genet Evol. 2014;21:227–43. doi: 10.1016/j.meegid.2013.10.023 24200587 10.1016/j.meegid.2013.10.023

[3] Dotzauer A, Kraemer L. Innate and adaptive immune responses against picornaviruses and their counteractions: An overview. World J Virol. 2012 6 12;1(3):91–107. doi: 10.5501/wjv.v1.i3.91 24175214 10.5501/wjv.v1.i3.91 PMC3782268

[4] CDC Epidemiology and Prevention of Vaccine-Preventable Diseases available The Pink Book: Course Textbook - 13th Edition (2015) athttps://www.cdc.gov/vaccines/pubs/pinkbook/hepa.html accessed on 2/2/2017

[5] Mellou K, Sideroglou T, Papaevangelou V, Katsiaflaka A, Bitsolas N, Verykouki E et al Considerations on the current universal vaccination policy against hepatitis A in Greece after recent outbreaks. PLoS One. 2015;10(1):e0116939 doi: 10.1371/journal.pone.0116939 25590132 10.1371/journal.pone.0116939 PMC4295885

[6] Bordi L, Rozera G, Scognamiglio P, Minosse C, Loffredo M, Antinori A et al Monophyletic outbreak of Hepatitis A involving HIV-infected men who have sex with men, Rome, Italy 2008–2009. J Clin Virol. 2012;54(1):26–9. doi: 10.1016/j.jcv.2012.01.009 22341552 10.1016/j.jcv.2012.01.009

[7] European Centre for Disease Prevention and Control. Hepatitis A outbreaks in the EU/EEA mostly affecting men who have sex with men–third update, 28 June 2017. Stockholm: ECDC; 2017.

[8] Field N, Cohen T, Struelens MJ, Palm D, Cookson B, Glynn JR et al Strengthening the Reporting of Molecular Epidemiology for Infectious Diseases (STROME-ID): an extension of the STROBE statement. Lancet Infect Dis. 2014;14(4):341–52. doi: 10.1016/S1473-3099(13)70324-4 24631223 10.1016/S1473-3099(13)70324-4

[9] Bruni R, Taffon S, Equestre M, Chionne P, Madonna E, Rizzo C et al Key Role of Sequencing to Trace Hepatitis A Viruses Circulating in Italy During a Large Multi-Country European Foodborne Outbreak in 2013. PLoS One. 2016;11(2):e0149642 doi: 10.1371/journal.pone.0149642 26901877 10.1371/journal.pone.0149642 PMC4764681

[10] COMMISSION IMPLEMENTING DECISION amending Decision 2002/253/EC laying down case definitions for reporting communicable diseases to the Community network under Decision No 2119/98/EC of the European Parliament and of the Council Official Journal of the European Union 27.9.2012

[11] Beebeejaun K, Degala S, Balogun K, Simms I, Woodhall SC, Heinsbroek E et al Outbreak of hepatitis A associated with men who have sex with men (MSM), England, July 2016 to January 2017. Euro Surveill. 2017;22(5):pii = 30454. 10.2807/1560-7917.ES.2017.22.5.30454 PMC5388117 28183392

[12] Freidl GS, Sonder GJ, Bovée LP, Friesema IH, van Rijckevorsel GG, Ruijs WL et al Hepatitis A outbreak among men who have sex with men (MSM) predominantly linked with the EuroPride, the Netherlands, July 2016 to February 2017. Euro Surveill. 2017;22(8):pii = 30468. http://dx.doi.org/10.2807/1560-7917.ES.2017.22.8.30468 10.2807/1560-7917.ES.2017.22.8.30468 PMC5356436 28251892

[13] GeoDemo Demography in figures available at http://demo.istat.it/index_e.html accessed on 31/01/2016

[14] Kulldorf M SaTScanTM User Guide 2015 availabe at https://www.satscan.org/ accessed on 31/01/2017

[15] European Centre for Disease Prevention and Control. Sexually transmitted infections in Europe 2013. Stockholm: ECDC; 2015.

[16] Werber D, Michaelis K, Hausner M, Sissolak D, Wenzel J, Bitzegeio J et al Ongoing outbreaks of hepatitis A among men who have sex with men (MSM), Berlin, November 2016 to January 2017 –linked to other German cities and European countries. Euro Surveill. 2017;22(5):pii = 30457. 10.2807/1560-7917.ES.2017.22.5.30457 PMC5388120 28183391

[17] Gozlan Y, Bar-Or I, Rakovsky A, Savion M, Amitai Z, Sheffer R et al Ongoing hepatitis A among men who have sex with men (MSM) linked to outbreaks in Europe in Tel Aviv area, Israel, December 2016 -June 2017. Euro Surveill. 2017 7 20;22(29). pii: 30575. doi: 10.2807/1560-7917.ES.2017.22.29.30575 . 28749336 10.2807/1560-7917.ES.2017.22.29.30575 PMC5532962

[18] Freidl GS, Sonder GJ, Bovée LP, Friesema IH, van Rijckevorsel GG, Ruijs WL et al Hepatitis A outbreak among men who have sex with men (MSM) predominantly linked with the EuroPride, the Netherlands, July 2016 to February 2017. Euro Surveill. 2017 2 23;22(8). pii: 30468. doi: 10.2807/1560-7917.ES.2017.22.8.30468 ; PubMed Central PMCID: PMC5356436. 28251892 10.2807/1560-7917.ES.2017.22.8.30468 PMC5356436

[19] Lopalco PL, Malfait P, Menniti-Ippolito F, Prato R, Germinario C, Chironna M et al Determinants of acquiring hepatitis A virus disease in a large Italian region in endemic and epidemic periods. J Viral Hepat. 2005;12(3):315–21. doi: 10.1111/j.1365-2893.2005.00593.x 15850473 10.1111/j.1365-2893.2005.00593.x

[20] Chironna M, Prato R, Sallustio A, Martinelli D, Tafuri S, Quarto M et al Hepatitis A in Puglia (South Italy) after 10 years of universal vaccination: need for strict monitoring and catch-up vaccination. BMC Infect Dis. 2012;12:271 doi: 10.1186/1471-2334-12-271 23098290 10.1186/1471-2334-12-271 PMC3527327

[21] La Rosa G, Libera SD, Iaconelli M, Ciccaglione AR, Bruni R, Taffon S et al Surveillance of hepatitis A virus in urban sewages and comparison with cases notified in the course of an outbreak, Italy 2013. BMC Infect Dis. 2014 7 29;14:419 doi: 10.1186/1471-2334-14-419 25074676 10.1186/1471-2334-14-419 PMC4122772

[22] European Centre for Disease Prevention and Control. Hepatitis A virus in the EU/EEA, 1975–2014. Stockholm: ECDC; 2016 Available on http://ecdc.europa.eu/en/publications/Publications/hepatitis-a-virus-EU-EEA-1975-2014.pdf

[23] Gossner CM, Severi E, Danielsson N, Hutin Y, Coulombier D. Changing hepatitis A epidemiology in the European Union: new challenges and opportunities. Euro Surveill. 2015;20(16). pii: 21101. 10.2807/1560-7917.es2015.20.16.21101 25953274

[24] Capobianchi MR, Garbuglia AR, Agrati C, Rianda A, Noto P, Corpolongo A et al Molecular characterization of hepatitis A outbreak in the province of Rome, Lazio region, Italy, January-July 2013. Microbes Infect. 2014;16(4):362–6. doi: 10.1016/j.micinf.2014.01.003 24486185 10.1016/j.micinf.2014.01.003

[25] WHO position paper on hepatitis A vaccines–June 2012. Wkly Epidemiol Rec. 2012;87(28/29):261–76. 22905367

[26] Ministero della Salute Piano Nazionale Vaccini 2012–2014 avallarle online at http://www.salute.gov.it/imgs/C_17_pubblicazioni_1721_allegato.pdf

[27] Ministero della Salute Piano Nazionale Prevenzione Vaccinale 2017–2019 avallarle online at http://www.salute.gov.it/imgs/C_17_pubblicazioni_2571_allegato.pdf.

